# Emergency and Mental Health Nurses’ Perceptions and Attitudes towards Alcoholics

**DOI:** 10.3390/ijerph15081733

**Published:** 2018-08-13

**Authors:** Jesús Molina-Mula, Antonio González-Trujillo, Margarita Simonet-Bennassar

**Affiliations:** 1 Nursing and Physiotherapy Department, University of Balearic Islands, 07122 Palma de Mallorca, Spain; 2 A&E Department, Balearic Health System, 07500 Manacor, Spain; antonio_pizarra@hotmail.com (A.G.-T.); hortea1@gmail.com (M.S.-B.)

**Keywords:** alcoholism, health professionals’ attitudes, social perception, drug-dependents

## Abstract

Knowing professionals’ attitudes is the basis for the development of skills for dealing with drug dependence. These attitudes may affect patients’ clinical safety and the cost-benefit ratio of the interventions. The goal of this study was to assess emergency and mental health nurses’ attitudes and perceptions towards alcoholics. A multicenter prospective descriptive study was conducted in six hospitals with 167 emergency and mental health nurses. Nurses classified alcoholics as sick individuals, although there was a tendency to feel uncomfortable working with them. Results indicated that these professionals had a rejecting attitude towards moderate alcohol consumption. It found that there was a significant association between the attitude of the nurse and gender, with the degree of rejection towards the alcoholic being higher in men than in women, and with less punitive attitudes in professionals with 0 to 11 years of professional experience.

## 1. Introduction

At present, alcoholism is socially considered to be a growing public health problem that has an impact on families, cultural patterns, and the economy. The Help Foundation Against Drug Addiction (FAD) and several authors [1,2,3,4] believe that drug-dependent conditions are conditioned by habits, and that social groups try to justify its possible negative effects by relating consumption to inherited cultural patterns. All of these aspects impact and shape health professionals’ attitudes towards the phenomenon of drug dependence.

Due to the heterogeneity in the scientific literature, it is difficult to separate alcoholism from the perception of other drugs in general, especially those of legal use. In general, there are publications that refer only to health professionals’ perception of alcoholics. However, those articles that referred to drug addiction and not only to alcoholism, have been included in this article, due to the relevance of their findings.

In this paper, when reference is made to drug dependents, it is made in a more general sense, not distinguishing between alcoholism and alcoholics when it refers specifically to this type of consumer.

In this research, the analysis focused only on the nurses’ perception and attitudes towards alcoholics, because there is no validated scale to evaluate such perceptions on drug dependences in general and for alcoholics specifically.

## 2. Background

A thorough literature search was carried out from 2010 to 2011, and again in 2017, by the Balear Research Group on Drug Dependence Nursing (GIBED), consulting PubMed, IME, COCHRANE, CINAHL, EBSCOhost, IBECS, and PSICODOC databases. As a result, two broad thematic categories emerged: studies that described or determined the impact of health professionals’ attitudes towards drug dependence care; and studies that analyzed the different perceptions of these professionals towards drug dependence care.

The results of the review revealed several aspects: (a) individual beliefs, age, sex, ethnicity, and professed religion, influenced attitudes towards drug dependents [5,6,7,8]; (b) professionals’ attitudes differed according to different roles, socialization, and the type and nature of contact with these patients [9], (c) the institutions in which the professionals worked influenced the care provided [10], (d) professionals should abstract from their preconceived ideas when meeting addicts’ needs [11,12], (e) there was a lack of training in drug dependence [7,13] and there were scarce undergraduate and graduate curricula on these subjects, (f) it is necessary to change the training that is currently provided [14,15], to achieve early detection and to perform preventive interventions in this matter [15,16]; (g) nurses have been more historically present than other health professionals in providing care to drug addicts, which represents a cornerstone in this matter [6].

In addition, the reviewed articles [17] revealed some differentiation in professionals’ attitudes according to the drugs consumed. Some addressed drug abuse in general, and others clearly differentiated alcohol consumption. However, in general, there was evidence that professionals had difficulties in understanding that substance dependence could be considered as an illness, especially alcohol dependence [6]. Howard and Chung [6] indicated that a substantial minority of nurses continued to consider that the consumption of addictive substances was immoral, resulting from a weak and defective nature of the consumers. 

The phenomenon of drug dependence is increasingly being more frequently addressed in services such as emergency and psychiatry, with alcoholism being the most frequent, followed by cannabis, in these units. Indig et al. [18], Clarke et al. [4] and Anderson et al. [19] stated that many professionals working in these services did not have the confidence, knowledge, skills, and sense of full and proper responsibility for the management of patients who suffer from drug dependence-related problems.

For this reason, it is important to describe the distinction made by various studies regarding professionals’ attitudes depending on the service to which they belong, i.e., emergency or psychiatry [20,21]. Pinikahana et al. [22] indicated that mental health professionals had non-discriminatory attitudes towards drug dependents, and did not consider that these patients should be excluded from the treatments after several relapses. This author does not make a clear distinction between different types of drugs. On the other hand, Kelleher and Cotter [23] found that emergency professionals considered drug dependents with multiple relapses to be intractable patients, in particular, chronic alcoholic patients who constantly went to the emergency room.

There is not enough scientific evidence on how health professionals’ attitudes impact on the quality of health care. Most studies have only described the different types of professionals’ attitudes and behaviors, but not their influence on the types of health care [17,20,21]. In addition, the literature is very scarce and there are no conclusive studies that identify the factors present in our health context.

Additionally, it has been found that there is a significant lack of validated instruments for the assessment of health professionals’ attitudes in our environment to describe and associate attitude types with the quality of health care provided [19,23].

Therefore, the goal of the present study was to assess nurses’ attitudes and perceptions in emergency and mental health services towards alcoholic patients, using the Seaman–Mannello scale. The study focused on alcoholic patients for two reasons: alcoholism is the principal drug dependence in the population that is treated in emergency and psychiatric units; there is a validated scale to assess nurses’ perceptions and attitudes towards alcoholism.

## 3. Methods

This was a multicenter, prospective, descriptive, and cross observational study that was conducted from March to October 2015, based on nurses in services with a high prevalence of care for people who were dependent on addictive substances, such as emergency, short stay, and psychiatry units. Since the total number of nurses in these services was easy to manage, all of the nurses working in the emergency and mental health services of five hospitals in the Balearic Islands, Spain, were selected, with a 20% adjustment for possible losses during follow-up. It included all nurses who had at least six months of professional experience and six months of service, by consecutive non-probabilistic sampling.

An anonymous letter was delivered to each nurse, with a questionnaire to collect demographic data, informed consent, and a Seaman–Mannello scale to measure nurses’ perceptions and attitudes towards alcoholism and alcohol consumption.

The Seaman–Mannello scale is a discrete quantitative scale, which is categorized, created, and validated by its authors in Buffalo (Nueva York) and later translated into Portuguese in Brazil by Pillon. A back-translation of the Portuguese version into Spanish was carried out by Villar to apply it in Bolivia, and it was later validated in its Spanish version for its use in Colombia by León and Vargas [24]. It consists of 30 items grouped into five subscales, to which the participants had to respond, according to their level of agreement or disagreement using a five-point Likert scale. It excluded the title of each subscale, and renumbered the questions from one to thirty, modifying and adapting the original questionnaire to facilitate participants’ comprehension.

Five subscales had six items each, which enabled the evaluation of five dimensions that related knowledge and attitudes, such as (1) the inclination in relation to therapy vs punishment, (2) personal/professional satisfaction in their work with alcoholics, (3) the inclination to identify their ability to help alcoholic patients, (4) the perceptions of personal characteristics of alcoholics, and (5) personal attitudes towards drinking. 

The Seaman–Mannello scale generated a Cronbach’s alpha of 0.693 for items that referred to the personal satisfaction of a professional working with alcoholics, 0.691 for the perception of dependence, and 0.775 for attitudes towards alcohol consumption. The factor analysis explained most factors up to 76% [24].

The study analyzed how the different variables behaved, using the SPSS Statistics software package (version 21.0; SPSS Inc., Chicago, IL, USA). It carried out a descriptive analysis of the studied population’s sociodemographic information using univariate analysis, considering the measures of central tendency (mean and standard deviation) for the quantitative variables, and the frequency distribution for the qualitative variables.

Subsequently, it analyzed the 30 questions of the Seaman–Mannello scale and its five subscales, attributing one and two points for unfavorable answers; three for the intermediate category; and four and five points for favorable categories. From the data obtained, it calculated the means, and this result was interpreted according to the recommendations of the instrument’s authors.

In addition, it included a bivariate analysis to assess the possible association between the subscales and the different variables, using a chi-square test for the dichotomous qualitative variables, and a Student’s *t*-test for the quantitative variables. The level of statistical significance considered was *p* < 0.05.

The study project was approved by the Research Ethics Committee of the Balearic Islands (IB3070/15PI) and the research commissions of the health centers where the research was conducted.

The study was funded by the Balearic Islands Nursing Official School. It met all the current ethical and legal principles, and the participants were informed correctly and signed a specific informed consent form before engaging in the present study.

## 4. Results

The studied population was composed of 257 nurses, from which it obtained 167 questionnaires, resulting in a loss of 35.02% (Table 1).

The analysis of the results obtained with the Seaman–Mannello scale was based on five subscales: subscale I—behaviors towards alcoholism, suggesting a dichotomy between therapy and punishment; subscale II—personal/professional satisfaction in working with alcoholics; subscale III—tendency to identify oneself with the ability to help alcoholic patients; subscale IV—perceptions towards alcoholics’ personal characteristics; and subscale V—nurses’ attitudes towards alcohol consumption.

Table 2 shows grouped descriptive statistics, taking into account that a mean that was lower than 12 points represented an unfavorable response; between 12 and 18 points represented a neutral positioning of the participants, and a mean greater than 18 points implied agreement or being in favor of the attitudes or premises of the scale.

However, each question and subscale was described in a disaggregated manner to better understand the results. Likewise, in the case of the grouped results, a mean lower than 2 points represented disagreement, 3 represented neutrality, and higher than 3 points represented agreement (Table 3).

It was observed in subscale I that 80.1% (130) of the nurses agreed or totally agreed that the lives of alcoholics were not pleasant, and that 61.9% (99) considered that alcoholics had very poor health. It is worth noting that only 5.3% (9) did not consider that such patients should receive psychiatric treatment, and 20.5% (18) were neutral in this regard.

An important result was that 76% (130) of the nurses considered alcoholics to be ill individuals. The mean values indicated that nurses agreed that alcohol-dependent patients should be treated as if they suffered from any other pathology.

The previous data should be contrasted with the results obtained in subscale II, that referred to nurses’ willingness to work with alcoholic patients. The means of the different items were mostly below 3 points, which represented dissatisfaction or dislike related to working with alcoholic patients. The results indicated that 42.7% (73) and 30.4% (52) did not feel better nor comfortable working with alcoholics, respectively, evidencing a neutral positioning by 42% (72) and 42.7% (73) concerning the two issues mentioned above. 

Therefore, although they considered alcoholics to be ill individuals, there was a tendency not to feel comfortable working with them. Even though the nurses did not perceive alcoholism as a taboo and accepted alcoholic patients as users, this pathology did not make nurses feel comfortable to work with such patients.

Subscale III assessed nurses’ tendency towards some considerations on alcoholics. There was a predominant neutral behavior regarding the fact that alcoholics would like not to be alcohol dependents. However, the greatest tendency was to disagree with the fact that alcoholics wanted to quit drinking, or that they respected their families. Only 4.1% (7) partially agreed, and no one totally agreed. There was no clear positioning with respect to considering that alcoholics could be treated, even if they did not want to quit drinking (22.8% (39) disagreed, 24.6% (42) were indifferent, and 39.2% (67) agreed).

The perceptions towards alcoholics’ personal characteristics were analyzed in subscale IV. Nurses’ neutral positioning was characterized by considering alcoholics as sensitive individuals with an inferiority complex and emotional difficulties, who consumed alcohol to escape from other problems.

Finally, subscale V assessed the nurses’ attitudes towards alcohol consumption. Negative personal attitudes towards alcohol consumption were predominant. It was observed that 44.5% (76) of the nurses disagreed that moderate alcohol consumption was beneficial to health, and 46.2% (79) disagreed that it was good. The results indicated that nurses had a rejecting attitude towards moderate alcohol consumption. They did not consider that this habit was healthy nor harmless at all, although there was a disparity of opinions regarding the belief that alcohol consumption made individuals weak.

A bivariate analysis was performed between each Seaman–Mannello subscale, relating the level of knowledge, sex, and preference for working with drug dependence, having received continuing training in drug dependence, and the length of service, which was not significant, according to the chi-square test. It is worth noting that there was a very weak Pearson’s correlation between age and the professionals’ attitudes towards alcohol consumption, as indicated by the data obtained in subscale V (*r* = 0.224; *p* = 0.04). It was not possible to perform a separate analysis of each unit, because the sample in the psychiatric unit was small.

In addition, a significant association (Table 4) was found between the attitude of the nurse and gender, with the degree of rejection towards the alcoholic being higher in men than in women (*t-*student *=* 2352; *p =* 0.02; CI_95%_ [0.43–4.49]), between the full scale (*t-*student *=* 2295; *p =* 0.023; CI_95%_ [5.41–43.64]) and the subscales II (*t-*student *=* 2462; *p =* 0.015; CI_95%_ [0.44–8.71]) and III (*t-*student *=* 3065; *p =* 0.003; CI_95%_ [0.85–10.06]), with less punitive attitudes in professionals with 0 to 11 years of professional experience in mental health or emergency services (Chi Squared *=* 16,792; *p =* 0.002; df = 5). Curiously, in our study there was no association between the assessment of the knowledge level and the attitudes measured in SM scale.

## 5. Discussion

The literature emphasizes that nurses influence the promotion of patients’ health and their treatment compliance, especially when nurses’ attitudes towards the patients are characterized by empathy, acceptance, and respect [15]. Some studies have concluded that health professionals’ attitudes are a determining factor in providing appropriate care to drug-dependent patients.

A study conducted by Carroll [9] showed a correlation between the degree of motivation and the resultant health professionals’ attitudes when working with drug dependents. Those professionals who chose to work with these patients had more positive attitudes than those professionals who had the patients imposed on them. Therefore, professionals who had previously worked with drug-dependent patients exhibited decreased fear and anxiety. If we compare these findings with the degree of professionals’ satisfaction indicated by our findings, we can observe professionals’ negative attitudes towards alcoholics and, therefore, less motivation for providing care [24]. This coincides with the result of perceptions about alcoholics and studies that did not distinguish the substance consumed.

Relevant similarities were found when we compared our data with those obtained by De Vargas and Labate [1,2,25] and Navarrete and Villar [26]. The common denominator in those two studies was personal rejection on the part of nurses towards moderate alcohol consumption, and the fact that they were prepared to help patients even if they continued drinking alcohol, because nurses considered that these patients should be treated.

In addition, nurses believed that the patients were sensitive individuals with severe emotional difficulties and an inferiority complex, whose lives were not pleasant and who lacked good physical health. Additionally, the studies mentioned above indicated that nurses were not comfortable when working with these patients, because they did not provide the satisfaction that other types of patients did; however, they accepted alcoholics as one more user.

The scores of subscale I obtained in the studies conducted by De Vargas and Labate [1,2,25] and ours showed high values, indicating that nurses believed that alcoholics were patients who were physically ill and needed medical treatment.

In the study conducted by De Vargas [2], subscale 2 obtained low scores, indicating feelings of dissatisfaction and displeasure on the part of the nurses, who at the time were working with individuals who exhibited problems related to alcohol consumption [23]. The present study also obtained a low score in the same subscale.

As well as in our study, the subscale V of the study conducted by Vargas and Labate [25] showed a low score. The authors divided these results into two categories: those related to nurses’ personal attitudes towards alcohol consumption; those related to alcoholics’ attitudes towards nurses. These findings were in line with the fact that nurses considered alcohol consumption as something dangerous and harmful in any amount, in addition to being morally wrong [17,25].

In general, different studies conducted in this field have shown that health professionals’ attitudes were very different, as can be observed in our results [2,26]. There was a predominance of more neutral or indifferent attitudes relating to the perceptions towards alcoholics and, especially, disagreement or dissatisfaction in working with these patients. This fact has was confirmed in a literature review conducted by Howard and Chung [6]. It is worth mentioning that these results are in line with our findings.

There are different instruments used for measuring nursing professionals’ attitudes when working with alcoholics. However, most studies have used the Seaman–Mannello scale [1,2,3,24,25,26] and, as previously mentioned, the results obtained by those studies were very similar to ours. All of those studies concluded that nurses were reluctant when they had to work with alcoholics. This reluctance has been confirmed in the score obtained in subscale II.

However, other studies have highlighted attitudes that have not been found in our study, such as the research conducted by Rassol, Villar, Carraro and Lopes [27] with nursing students, in which most of the participants considered alcoholics to be boring, annoying, disgusting, deceptive, false, and liars. These students described the patients as reckless and irresponsible individuals, who did not want to recover, who were cowards when they had to fight for themselves, and who did not undertake the treatments [27].

Although no association was found between sociodemographic variables, knowledge, and experience in the scores of the scale, Howard and Chung [6] observed that better educated, younger nurses, with higher graduation rates and less professional experience had better knowledge and more positive attitudes towards drug dependents than their colleagues. Moody [28] concluded that middle-class nurses with more authoritarian attitudes were less willing to work with alcoholic patients. In addition, in so far as patients were seen by nurses as aggressive individuals who distorted their work, their professional attitudes became more intolerant, and they had low expectations of the care provided.

Our findings are in line with the tendency that was highlighted in the studies conducted by Cooper [29] and Carroll [9], who observed a gradual abandonment of nurses’ conception that regarded addicts as patients with low self-esteem and that should not be considered as other patients. In this regard, it is worth noting a more positive attitude in male than in female nurses towards care provided to drug dependents, thus explaining the correlation found in our study between sex and health professionals’ attitudes towards alcohol consumption.

In addition to the biases involved in the application of any measuring scale, our study had the limitation of participation and sample size. We emphasized that, since the study had a descriptive design, the possible associations were found to have no validity to establish a causal relationship between the variables; however, they can encourage further studies with an analytical design intended to assess and contrast the respective hypotheses.

This study has the limitation of being a descriptive study, which did not allow the assumption of a cause and effect relationship between the associated variables. In addition, although the scale used is validated, it can present coherence problems, since it evaluates perceptions and attitudes in different moments and social contexts. Finally, it is highlighted that since the psychiatry unit is a service with fewer nurses, the sample obtained was low, thus not allowing comparison with the emergency unit.

## 6. Conclusions

The present study has described nurses’ perceptions and attitudes towards alcoholic patients. Analyzing the different dimensions of the scale used, it is concluded that these health professionals considered alcoholics as patients with poor physical health, emotional problems, undermined social relations, and in need of treatment. The nurses in our sample exhibited a certain dissatisfaction in working with alcoholics and preferred other types of patients. It was also observed that these professionals exhibited rejecting attitudes towards alcohol consumption, even in moderate amounts.

Therefore, it is necessary to conduct studies to assess the quality of health care provided by nurses to drug dependents in our healthcare environment and expand upon the impact of these attitudes on the care provided to these patients. Considering these results and relating them to the scientific evidence that determines which aspects or factors condition health professionals’ perceptions and attitudes, can affirm that they should be taken into account to provide quality care to drug dependents.

Ethnographic and phenomenological studies are required to provide deeper insight into the field of professionals’ social perceptions and attitudes towards drug dependence. This will enable us to learn about the phenomenon in greater depth and determine the reasons behind such attitudes and perceptions.

The information obtained in the present study allowed us to determine health professionals’ attitudes, thus providing a basis for the development of skills required to address drug dependence. These attitudes may affect patients’ clinical safety and the cost–benefit ratio of the interventions performed.

## Figures and Tables

**Table 1:** Distribution of the sample according to sociodemographic characteristics.

**Service**	**F**	**%**	**F**	**%**
	Valid		Losses	
Urgencies	126	75.45	83	32.30
Psychiatry	41	24.55	7	2.72
**Sociodemographic Characteristics**	**F**	**%**	**F**	**%**
	Men		Women	
Gender	44	27.3%	117	72.7%
	Spanish		Others	
Nationality	163	97.0%	5	3%
	Masters and others		Official Speciality	
Postgraduate Studies	66	36.8%	17	9.9%
	0–11 years		12–25 years	
Professional Experience	48	25.7%	120	62.7%
	Permanent		Non-Permanent	
Employment status	123	71.9%	5	2.9%
	Register Nursing		Management	
Professional Activity	161	96.4%	6	3.6%
	Yes		No	
Contact with Drug Dependences (last six months)	151	88.3%	18	10.5%

**Table 2:** Grouped descriptive statistics of the Seaman–Mannello scale.

		Complete Seaman–Mannello Scale	Subscale I	Subscale II	Subscale III	Subscale IV	Subscale V
No.	Valid	167	167	167	167	167	167
	Lost	4	4	4	4	4	4
Mean		85.00	21.33	15.88	16.29	17.20	14.27
Standard deviation		30.94	8.08	6.21	6.61	6.58	6.46
Minimum		0	0	0	0	0	0
Maximum		124	30	26	27	28	28

**Table 3:** Disaggregated descriptive statistics per response in the Seaman–Mannello scale.

	No.	Min	Max	Mean	Standard Deviation
Subscale I: Tendency towards the patient: therapy versus punishment					
01. The lives of alcoholics are not pleasant.	150	1	5	4.24	0.774
02. In general, alcoholics have poor physical health.	149	1	5	3.74	0.940
03. I think it is very painful that alcoholics usually suffer from delirium tremens.	148	1	5	3.29	1.071
04. Alcoholic patients need psychiatric help.	148	1	5	4.17	0.914
05. Alcoholics should receive medical treatment.	147	1	5	4.19	0.855
06. Alcoholism is an illness.	148	1	5	4.39	0.966
Subscale II: Personal/professional satisfaction in working with alcoholics					
07. I feel that I work better with alcoholic patients.	148	1	5	2.32	0.843
08. I prefer to work with alcoholics rather than other patients.	147	1	5	2.24	0.909
09. Alcoholics deserve a place in the hospitals just like other patients.	148	1	5	3.78	1.000
10. I do not think that my patients become angry if I discuss their excessive alcohol consumption with them.	146	1	5	2.92	0.921
11. I feel comfortable working with alcoholics.	149	1	5	2.79	0.843
12. I am not ashamed of speaking about alcoholism.	149	1	5	3.89	0.983
Subscale III: Tendency to identify oneself with the ability to help alcoholic patients					
13. Alcoholics are not only concerned with their happiness.	147	1	5	3.29	0.979
14. Alcoholics respect their families.	147	1	4	2.31	0.833
15. Alcoholics want to quit drinking alcohol.	147	1	5	2.86	0.911
16. Alcoholics who do not obey nurses’ orders also want to be treated.	147	1	5	3.42	1.072
17. Most alcoholics would like not to be addicted to alcohol.	147	1	5	3.42	0.891
18. I can help alcoholics even if they do not quit drinking alcohol.	148	1	5	3.20	1.073
Subscale IV: Perceptions towards alcoholics’ personal characteristics					
19. Alcoholics are sensitive individuals.	148	1	5	3.00	0.808
20. Alcoholics exhibit an inferiority complex.	148	1	5	3.21	0.767
21. Alcoholics started drinking alcohol due to other problems.	148	1	5	3.28	0.857
22. Alcoholics do not feel they are bad persons due to alcohol consumption.	148	1	5	3.14	0.744
23. Alcoholics are loners.	149	1	5	3.13	0.925
24. Alcoholics usually exhibit serious emotional difficulties.	148	1	5	3.64	0.850
Subscale V: Personal attitudes of health professionals towards alcohol consumption					
25. Moderate alcohol consumption can really bring benefits to persons’ health.	146	1	5	2.60	1.184
26. There is nothing wrong with moderate alcohol consumption.	149	1	5	2.56	1.042
27. Alcoholic beverages are harmless if drunk moderately.	149	1	5	2.58	1.054
28. Individuals should drink alcoholic beverages if they want to.	149	1	5	2.77	1.053
29. If used wisely, alcoholic beverages are not more harmful than non-alcoholic beverages for normal adults.	149	1	5	2.51	1.050
30. Alcohol consumption does not make normal individuals week or fools.	149	1	5	3.04	1.096

**Table 4:** Relationship between demographic data and the Seaman–Mannello Scale

	CI_95%_		*t-*Student	*p*
	Min	Max		
Gender				
Degree of rejection towards the alcoholic	0.43	4.49	2352	0.02
Seaman–Mannello Scale (Full)	5.41	43.6	2295	0.023
Seaman–Mannello Scale (Subscale II)	0.44	8.71	2462	0.015
Seaman–Mannello Scale (Subscale III)	0.85	10.1	3065	0.003
		**df**	**χ^2^**	***p***
0–11 Years of Professional Experience				
Less punitive attitudes in professionals		5	16,792	0.002
